# Modified Technique for Scleral-Sutured Fixation with the Double Knots Technique for Posterior Chamber Intraocular Lens: Short-Term Observation

**DOI:** 10.1155/2021/6697230

**Published:** 2021-02-27

**Authors:** Zhizhong Wu, Caijuan Liu, Yanhui Xu, Wei Dong, Zhimin Chen

**Affiliations:** Department of Ophthalmology, Hebei Eye Hospital, Xingtai, Hebei, China

## Abstract

**Purpose:**

To evaluate the short-term safety and efficacy of a novel approach of utilizing the 9-0 looped polypropylene suture with double knots buried into the scleral groove and the scleral tunnel to minimize the risk of the suture erosion and suture knot exposure.

**Design:**

Clinical-based retrospective study.

**Methods:**

Records of consecutive patients who had anterior vitrectomy and scleral-fixated posterior chamber intraocular lens (IOL) implantation between July 2018 and April 2020 with a minimum follow-up of 3 months were reviewed.

**Results:**

This study enrolled a total of 21 eyes from 20 patients (15 male). These patients had a mean age of 58.52 ± 8.55 years and were followed for an average of 1.08 ± 0.58 years postoperatively. Best-corrected visual acuity (BCVA) improved from a preoperative mean of 0.43 ± 0.41 logMAR to a significantly higher mean 3-month postoperative value of 0.09 ± 0.21 logMAR (*Z* = -3.35, *p* < 0.01). There were no statistical differences between the preoperative and postoperative corneal endothelial cell density (*p*=0.71). The postoperative complications included transient increased intraocular pressure in 5 eyes (24%). No other complications were detected during the follow-up.

**Conclusions:**

The modified technique proposed is a safe, effective, and reliable approach resulting in good visual outcomes. Our procedure might have the potential benefit to avoid suture-related complications in scleral-fixated IOL implantation. *Trial registration*. Retrospective case series study, not applicable.

## 1. Introduction

There are many reports of intraocular lens (IOL) implantation in the absence of capsular support. Representative techniques include anterior chamber IOL (AC IOL), iris-fixed IOL, and scleral-fixated IOL implantation via suture-based or suture-free intrascleral fixation [1–3]. Transscleral-sutured IOL fixation was first proposed by Malbran in 1986 [4] and is a relatively common surgical approach owing to the advantages such as not disrupting eye's anatomy and contacting with corneal endothelium. However, suture erosion, suture knots exposure, and recurrent dislocations remain to be the common late complications caused by suture breakage [5–7]. Efforts to prevent these complications have been employed including the rotating of suture knots into the eye, burying the suture ends into scleral tunnel, and covering suture ends with tenon's capsule, scleral flaps, or pockets [8–14]. The key issues related to this process are the appropriate selection of suture materials and suture knotting approaches. The use of 10-0 polypropylene sutures is becoming increasingly unpopular because they degrade over time, eventually leading to the suture slippage and the subluxation and dislocation of the IOL.

The purpose of this study was to evaluate the safety and efficacy of a novel approach of utilizing the 9-0 looped polypropylene suture with double knots buried into the scleral groove and the scleral tunnel to minimize the risk of the suture erosion and suture knot exposure.

## 2. Patients and Methods

### 2.1. Patients

Surgical outcomes of 21 eyes from 20 patients that underwent treatment using the 9-0 looped polypropylene suture to achieve IOL scleral fixation with double ball-shaped knots at the Hebei Eye Hospital between July 2018 and April 2020 were retrospectively reviewed. Patients with any underlying ocular pathology, namely, keratopathy, glaucoma, macular edema, and operated vitreoretinal surgery, were excluded to avoid any confounding factor. All patients were conducted by a single experienced surgeon (ZMC). This study was consistent with the Declaration of Helsinki.

Data from patients with *a* < 3-month postoperative follow-up or with incomplete operative or postoperative medical records were excluded from this study. Enrolled patients had complete records pertaining to their visual acuity (VA), slit-lamp photographs, and ultrasound biomicroscope (UBM) findings [15]. During their initial baseline visit, each patient was subjected to comprehensive ophthalmic evaluations of best-corrected visual acuity (BCVA), as well as intraocular pressure, lens status, previous surgeries, preexisting ocular pathologies, and history of ocular trauma [15]. The list of analyzed postoperative outcome measures comprised BCVA, corneal endothelial cell density, and complications. The following logMAR cutoffs were used for non-numeric VA [16]: able to count the fingers = 1.7 logMAR, able to detect the hand movement = 2.0 logMAR, light perception = 2.3 logMAR, and no light perception = 3.0 logMAR.

### 2.2. Surgical Techniques

**Figure 1:**
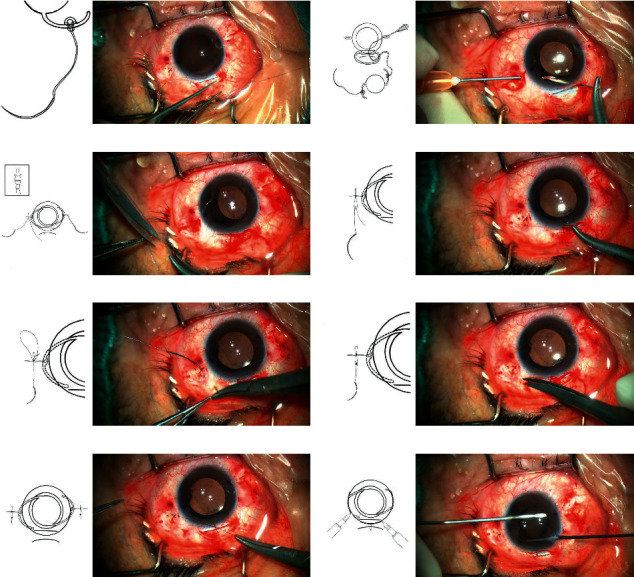
The process of transscleral-sutured IOL fixation. (a) Cow-hitch knots are created with the method that the long curved needle passes through the eyelet of one haptic of the IOL. (b) The curved needle was introduced through the corneal incision, docked into a 23-G needle introduced from the 2 o'clock and 8 o'clock scleral incision, and pulled out. (c) The first ball-shaped knot was created in 3-2-1 fashion. (d) The second ball-shaped knot was created in 3-2-2 fashion and tied about 3 mm away from the first knot. (e) The long needle with thread entered the other scleral lamina from the incision and sneaked 4-5 mm between the scleral layers. (f) The second thread knot was buried in the deep sclera. (g) The same procedures were performed for the opposite side and IOL centration adjusted. (h) The peripheral iris was excised at 1 o'clock with 23-gauge vitreoretinal surgery.

**Figure 2:**
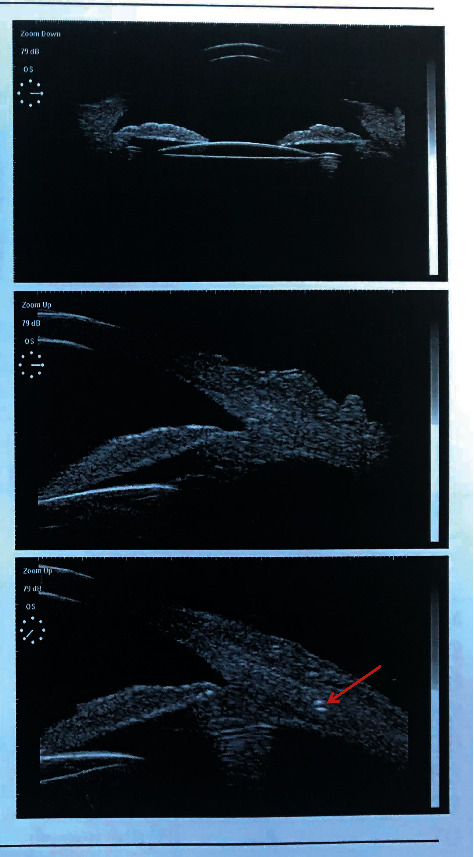
The typical UBM images in a patient 3 months after the transscleral-sutured IOL implantation (tunnel containing the sutures was clearly detected by UBM (red arrow)).

**Figure 3:**
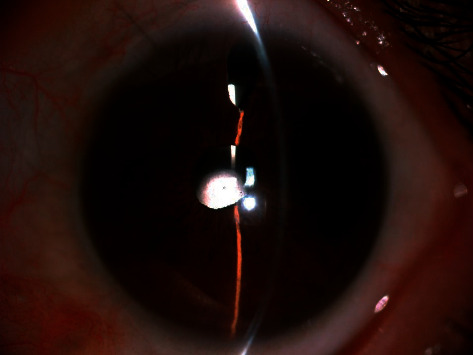
Postoperative 3-month follow-up anterior segment photograph of a 55-year-old woman who underwent the transscleral-sutured IOL implantation due to complicated cataract surgery.

The surgeries were performed under general anesthesia. All patients were operated on by the same surgeon, using a similar technique. All patients were subjected to anterior vitrectomy. All study subjects were implanted with a single-piece polymethyl methacrylate (PMMA) IOL with an optic diameter of 6.5 mm and overall diameter of 12.5 mm (EyeKon Medical, Inc). Fixating sutures were placed at 2 and 8 o'clock positions and 1 mm posterior to the limbus, through the ciliary sulcus. Intraocular volume was maintained via viscoelastic agents throughout the procedures. All scleral fixation sutures were made of 9-0 looped polypropylene suture (Mani, Inc, model: 2452L, Japan). First, the surgeon opened the conjunctiva at the superior position to make a 6.0 mm scleral tunnel incision. Then, two conjunctiva snips were performed at 2 o'clock and 8 o'clock, and two radial incisions of partial-thickness scleral were made at the conjunctiva snips 1 mm from the limbus. Viscoelastic was injected into the anterior chamber to maintain volume. Cow-hitch knots were than created with the method that the long-curved needle passed through the eyelet of the haptic of the IOL, allowing the polypropylene suture to tie to the haptic of the IOL (Figure 1). The curved needles were introduced through the scleral tunnel incision and subsequently inserted into the 23-gauge needle hole (Figure 1). The needle was externalized through radial scleral incision and detached from 23 G needle leaving 9-0 looped polypropylene suture outside the scleral wound. After implanting the intraocular lens (IOL) in the posterior chamber, the IOL was secured by slowly pulling the external sutures to introduce the haptics into the ciliary sulcus. One string of the looped suture was then cut at the end of the needle (Figure 1). The needle suture was inserted parallel to the limbus through the scleral layer on one side of the radial scleral incision, and then, this intrascleral pass was repeated once in the respective opposite direction, finally resulting in a U pattern suture. After the needle out at the radial scleral incision, the first ball-shaped knot was created in 3-2-1 fashion with the previous cut thread (Figure 1). The second ball-shaped knot was then created in 3-2-2 fashion and tied about 3 mm away from the first knot (Figure 1). After cutting the free suture (without needle), the long needle with thread entered the other scleral lamina from the radial scleral incision and sneaked 4-5 mm between the partial-thickness scleral layers (Figure 1). Finally, the second thread knot was buried in the partial-thickness sclera (Figure 1), and the suture was cut at the outlet of the scleral tunnel. The same procedures were performed for the opposite side and IOL centration adjusted (Figure 1). After the anterior chamber was injected with carbaciline injection and the pupil was constricted, the peripheral iris was excised at 1 o'clock with 23-gauge vitreoretinal surgery (Figure 1). The conjunctiva was closed by an eye hemostatic device, and the superior scleral tunnel wound was closed with one 10-0 monofilament nylon suture. Tobramycin dexamethasone eye ointment was applied to the conjunctival sac at the end of the procedure. Postoperatively, topical antibiotic/steroid drops were given four times a day for 2-4 weeks. Figures 2 and 3 show the postoperative status of one patient in our study.

### 2.3. Statistical Analyses

Data were tested for normal distribution using the Kolmogorov–Smirnov test. Descriptive statistics are the mean ± standard deviation (SD) for normally variables and median values/interquartile range for non-normally variables. *p* values of <0.05 were considered statistically significant. All statistical analyses were performed using IBM SPSS Statistics Version 22.

## 3. Result

### 3.1. Basic Patient Characteristics

This study enrolled a total of 21 eyes from 20 patients (5 female and 15 male). Patient baseline characteristics including their surgical indications, lens status, and postoperative data are compiled in Table 1. These patients had a mean age of 58.52 ± 8.55 years and were followed for an average of 1.08 ± 0.58 years postoperatively. Causes of inadequate capsular support in these patients included subluxated lens, complicated cataract surgery, traumatic cataract, dislocated previously implanted posterior chamber IOL, or aphakia with need for secondary IOL.

### 3.2. Postoperative Best-Corrected Visual Acuity

Best-corrected visual acuity (BCVA) improved from a preoperative mean of 0.43 ± 0.41 logMAR to a significantly higher mean 3-month postoperative value of 0.09 ± 0.21 logMAR (*Z* = -3.35, *p* < 0.01). There were no statistical differences between the preoperative and postoperative corneal endothelial cell density (*p*=0.71).

### 3.3. Postoperative Complications

The postoperative complications included transient increased intraocular pressure in 5 eyes (24%), which was treated with the antiglaucomatous drops or anterior chamber puncture. Subsequent surgical intervention was not required. No other complications (e.g., cystoid macular edema, retinal detachment, endophthalmitis, iris capture of the IOL, and significant IOL tilt) were detected during the follow-up. Postoperatively, ultrasound biomicroscopy (UBM) showed the IOL was well centered in all cases and the suture was well tucked into the scleral wall (Figures 2 and 3).

## 4. Discussion

Many techniques of transscleral fixation of posterior chamber intraocular lens have been developed [17]. The focus is on preserving the integrity of the anterior chamber, reducing iridic contact and avoiding the damage of the ocular anatomy. Compared with AC IOL, retro-iris implantation is the main advantage of scleral-fixated PC IOL. Therefore, damage of the endothelial cells and the angle structures can be avoided [1, 17, 18].

Recently, more and more research has been done on the sutureless intrascleral IOL fixation [19–22]. Intraoperative posterior IOL dislocation, haptic kink, and fracture are possible complications during haptic externalization [22, 23]. To date, it is noteworthy that none of the 3-piece IOL haptics was designed for an intrascleral implantation. Due to the above limitations of the sutureless intrascleral IOL fixation, the transscleral suture-mediated IOL fixation is still a relatively common surgical approach. However, complications of this approach when it is conducted in concert with stitching methods include suture-induced erosion, suture exposure, and suture rupture-related IOL dislocation. Multiple studies have shown that method burying the suture ends into scleral tunnel may be a better way to reduce the occurrence of the suture-induced inflammation, suture erosion, and the suture exposure [14, 24]. In this study, an improved suture method was used: first, a radial scleral incision was used for the needle exit, which is simple to make. As the incision heals naturally, the suture knot could be buried in the incision. Secondly, after the needle was drawn out, the loop thread was cut off, and the side connected with the needle traveled back and forth between the scleral layers and then sutured the other side to the scleral radial incision, effectively avoiding the cutting effect of the suture tension on the sclera and the suture slippage. The second knot was located about 3 mm away from the previous knot, and it was buried between the layers of the sclera (Figure 1). This may effectively avoid the production of scleral flaps, and sutures were better buried, which minimized the risk of suture exposure. At the same time, there is less risk of slippage in the presence of double spaced knots, which have a certain protective effect on the first suture knot.

Optimal suture selection has been suggested as a viable approach to improve patient outcomes. Polypropylene suture has long been used in ophthalmic surgery because of its advantages of stress resistance, stability, and biocompatibility [25]. A growing body of research suggests that the use of 9–0 polypropylene suture can minimize or eliminate the risks of complications associated with 10–0 polypropylene sutures such as suture degradation and secondary intraocular lens shift [26–28] due to its 60% greater tensile strength. For these reasons, looped polypropylene sutures attached with curved 9-0 needle were introduced into our technique to strengthen the haptic fixation. In current study, the 9-0 polypropylene suture directly passed through the round hole of the intraocular lens loop to form a cow-hitch knot, without suture, and almost no contact with surgical machinery, which effectively avoids the tension and clamping of the suture thread in the intraocular segment and the subscleral segment. Recent research has shown that the fracture of intraocular segment sutures accounts for quite a large proportion [28]. Although there are no long-term data to support this theory, we expect that this technique should minimize the risk of IOL dislocation from late suture-related complications.

In our study, by means of a relay, the 23 needles first entered the anterior chamber at the marked position, thus leading to the stitches, which helped simplify the identification of sclerotomy sites and provided control over the insertion angles. This method may effectively reduce the incidence of IOL tilt and eccentricity after surgery and decrease the number and time of surgical instruments entering the eye, thereby reducing the occurrence of a series of complications such as endophthalmitis and intraocular hemorrhage [15, 27]. Postoperative best-corrected visual acuity was improved in most of patients of current study (*p* < 0.01), and no serious complications occurred except for a few cases of transient intraocular pressure increase during the follow-up period. All patients released a small amount of aqueous humor through several side corneal incisions, and the intraocular pressure returned to normal levels. Residual viscoelastic agent in the eye may be the main cause of postoperative intraocular pressure increase.

This study has some limitations. First, the sample size is too small. Future studies are required with the larger sample and longer follow-up periods to understand the long-term implications and stability of this modified surgical technique. Second, in the current study, a large corneoscleral incision was required for implantation of nonfoldable PMMA lens, which may result in postoperative astigmatism and other complications [29].

In conclusion, the modified technique proposed is a safe, effective, and reliable approach resulting in good visual outcomes. This treatment can achieve excellent IOL stability and results in low rates of intraoperation and postoperation complications. However, our study was based on a short-term follow-up period. A larger sample size and longer follow-up periods of the surgical results are required to verify the safety and efficacy of our modified technique.

## Figures and Tables

**Table 1:** Baseline characteristics and postoperative data.

Characteristics	Value
**Eyes/patients (*n*)**	21/20
**Mean age (y)** ± **SD**	58.52 ± 8.55
**Male/female sex (*n*)**	15/5
**Diagnosis, *n* (%)**	
Subluxated lens	7 (33)
Complicated cataract surgery	2 (10)
Traumatic cataract	1 (5)
Dislocated previously implanted posterior chamber IOL	7 (33)
Aphakia with need for secondary IOL	4 (19)
**Mean follow-up (y)** **±** **SD**	1.08 ± 0.58
**Mean baseline logMAR BCVA** **±** **SD**	0.43 ± 041
**Mean logMAR BCVA at 3 mo** **±** **SD**	0.09 ± 0.21
**Mean baseline ECD (cells/mm** ^2^ **)** **±** **SD**	2566 ± 653
**Mean ECD at 3 mo (cells/mm** ^**2**^ **)** **±** **SD**	2524 ± 668

SD = standard deviation; IOL = intraocular lens; mo = month; *n* = number; y = year; BCVA = best-corrected visual acuity; logMAR = logarithm of the minimum angle of resolution; ECD = endothelial cell density.

## Data Availability

The data used for the analysis are available from the corresponding author upon reasonable request.

## Supplementary Materials

Supplementary Materials Supplemental digital content: video. Modified technique for scleral-sutured fixation with the double knots technique for posterior chamber intraocular lens (https://1drv.ms/u/s!athd9opzzcr6aw80lxngii0qzm0/).
