# Supplementary material for: Modified Technique for Scleral-Sutured Fixation with the Double Knots Technique for Posterior Chamber Intraocular Lens: Short-Term Observation
Source: J Ophthalmol. 2021 Feb 27;2021:6697230. doi: 10.1155/2021/6697230 (PMC7937454; doi:10.1155/2021/6697230)
Supplement: Supplementary Materials — Supplemental digital content: video. Modified technique for scleral-sutured fixation with the double knots technique for posterior chamber intraocular lens (https://1drv.ms/u/s!athd9opzzcr6aw80lxngii0qzm0/). [file 6697230.f1.docx]

https://1drv.ms/u/s!AtHd9OpzzCr6aW80LXNGiI0QZm0
